# Is Gangliocytic Paraganglioma Designated as a Subtype of Composite Paragangliomas and Originated From Pancreas Islet? A Case Report and Review of Literature

**DOI:** 10.3389/fendo.2022.847632

**Published:** 2022-03-01

**Authors:** Jing Li, Lu-Ping Wang, Pei-Shuang Zhu

**Affiliations:** Department of Pathology, The 7th Medical Center, Chinese PLA General Hospital, Beijing, China

**Keywords:** gangliocytic paraganglioma, duodenum, immunohistochemistry, progesterone receptor, paraganglioma-ganglioneuroma

## Abstract

Gangliocytic paraganglioma (GP) is quite rare, and origin and entity remain to be elucidated. A 51-year-old man presented with GP as a sessile polyp with a smooth surface that measured about 1 cm in diameter in the descending portion of duodenum. Pathological examination displayed that a neoplasm was predominantly located in the submucosa and infiltrated mucosa focally. The tumor consisted of epithelioid, ganglion-like, and spindle cells admixing in a haphazard way. The epithelioid cells resembled paraganglioma in cytological and architectural features. The ganglion-like cells were scattered and merged with the bland spindle cells in fascicular clusters, which resembled ganglioneuroma. Synaptophysin (Syn), microtubule-associated protein-2 (MAP-2), and chromogranin A (CgA) were positive in the epithelioid and ganglion-like cells in variety, and neurofilament (NF) staining highlighted the ganglion-like cells. S-100 and SOX-10 were positive in the spindle cell proliferation and around the epithelioid cells. Progesterone receptor (PR) was positive in the epithelioid cells. The polyp was resected, and no adjuvant therapy was given. The patient remained with no recurrence in 2 years’ follow-up. Origin of GP is presumed to be related to pancreas islet. GP is distinguished from neuroendocrine tumor (NET) G1 and designated as paraganglioma-ganglioneuroma, a kind of composite paragangliomas.

## Introduction

Paraganglioma (PGL) always involves the extra-adrenal ganglions among the sympathetic or parasympathetic chain. Gangliocytic paraganglioma (GP), a distinct type of PGL, is quite rare, and only up to 300 cases have been reported since it was first described in 1957. Origin and entity of GPs remain to be elucidated. We experienced a case of GP in the descending portion of the duodenum. Herein, we presented the case and discussed with review of literature.

## Case Description

**Figure 1:**
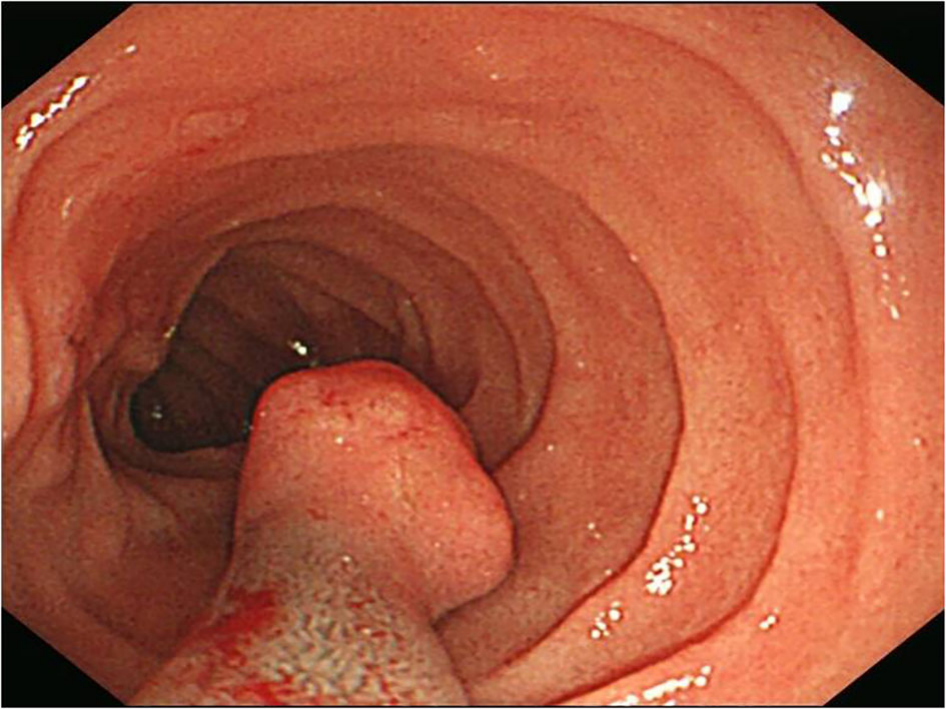
A sessile polyp human melanoma black-45 (HMB45) measured about 1 cm in diameter with a smooth surface was found in gastrointestinal endoscopy.

A 51-year-old man had presented with abdominal discomfort for several years. Physical and experimental examinations revealed no significant differences. Gastrointestinal endoscopy was performed, and a sessile polyp with a smooth surface that measured about 1 cm in diameter was found in the descending portion of the duodenum (**Figure 1**). The lesion was excised then.

**Figure 2:**
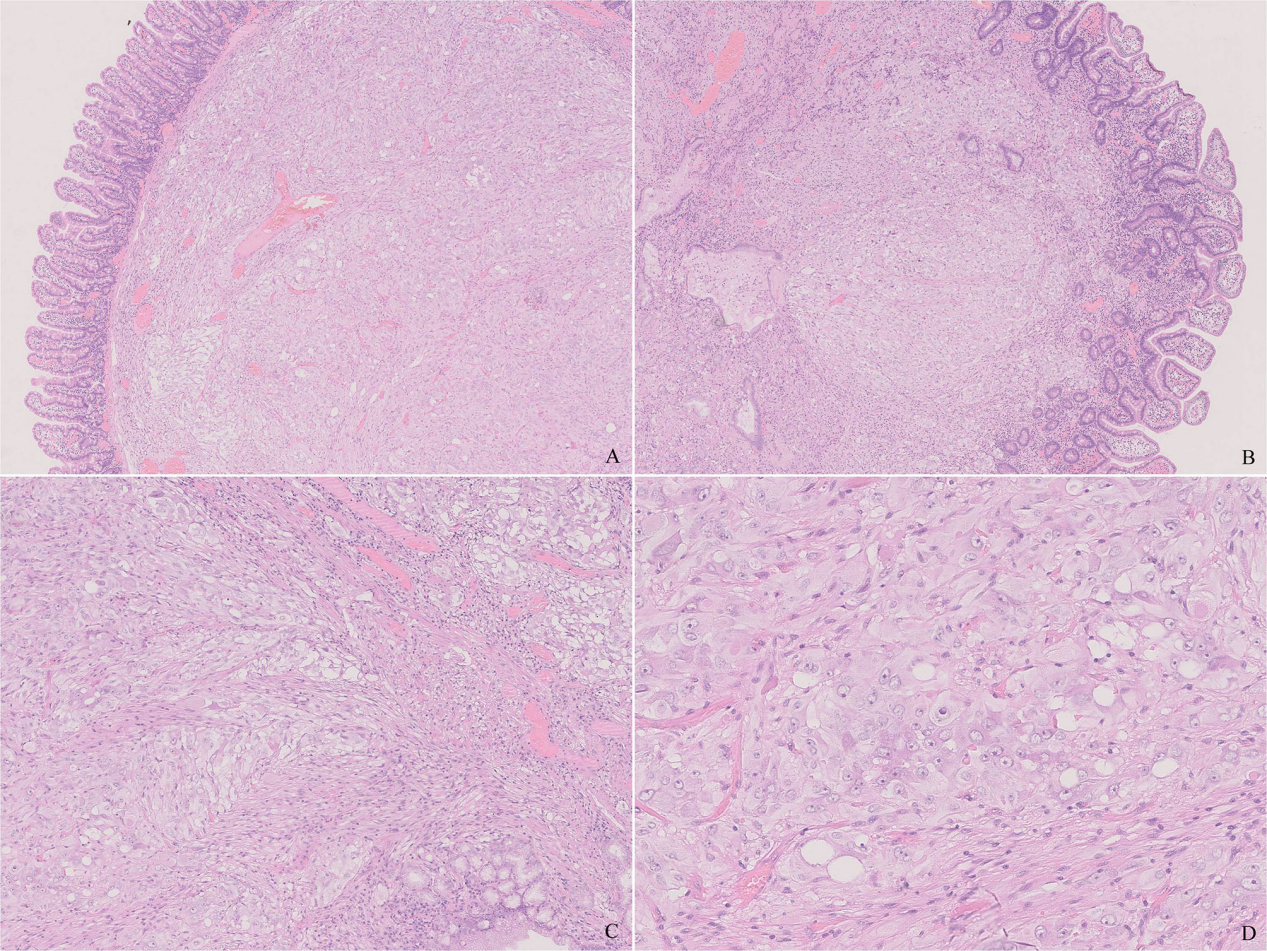
Histology of the tumor. **(A)** The tumor was well-circumscribed and non-encapsulated and located in the submucosa. **(B)** The tumor infiltrated the lamina propria focally. **(C)** The tumor was composed of epithelioid, ganglion-like, and spindle cells. Note the muscularis mucosae in the upper right that are different from the neoplastic spindle cells. **(D)** The epithelioid cells have abundant eosinophilic cytoplasm and a round nucleus. Note a ganglion-like cell in the center, which was larger in shape, and a nucleus with prominent eccentric nuclei. [**(A, B)**, H&E ×20, **(C)**, H&E ×100, **(D)**, H&E ×400].

Microscopically, the tumor was predominantly located in the submucosa, infiltrated the lamina propria focally, and covered with duodenal mucosa (**Figures 2A, B**). It was composed of epithelioid cells, ganglion-like cells, and spindle cells (**Figure 2C**). The epithelioid cells were large in round or polygonal shape and arranged in nest or zellballen pattern. They had abundant eosinophilic cytoplasm and a round nucleus with inconspicuous nucleoli. Some were larger with granular cytoplasm and vesicular nuclei with prominent nucleoli. The ganglion-like cells were even larger with eosinophilic cytoplasm and out-standing eccentric vesicular nuclei and were always scattered and not easily distinguished from the epithelioid cells (**Figure 2D**). Both types of cells displayed mild cellular pleomorphism yet did not display mitosis. The spindle cells were bland and arranged in fascicular clusters. The three proportions were admixed in a haphazard way. Some distorted and enlarged glands were entrapped in the lesion. The tumor did not display inflammation, necrosis, and calcification.

**Figure 3:**
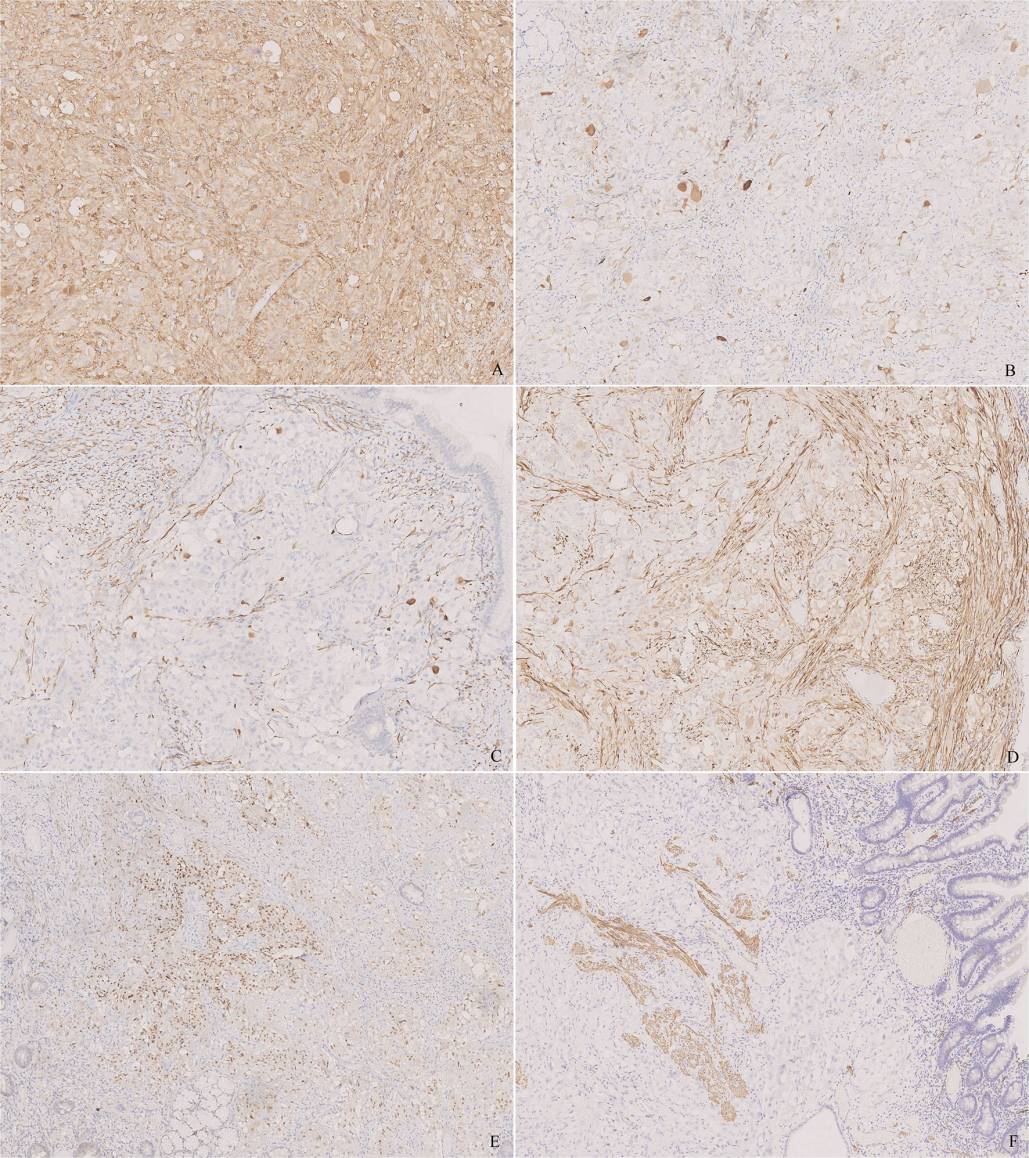
Immunohistochemistry of the tumor. **(A)** Synaptophysin (Syn) was diffusely positive for epithelioid cells and highlighted the ganglion-like cells. **(B)** Chromogranin A (CgA) was positive for epithelioid cells and highlighted the ganglion-like cells, but the number of positive cells was less than that in Syn. **(C)** Neurofilament (NF) stained some ganglion-like cells and neoplastic spindle cells. **(D)** S-100 stained the sustentacular cells around the epithelioid cells and the neoplastic spindle cells. **(E)** Progesterone receptor (PR) was positive in some epithelioid cells. **(F)** Desmin stained the muscularis mucosa, which displayed that the tumor infiltrated the lamina propria. [**(A–F)**, ×100].

Immunohistochemical study showed an extremely low index of Ki-67. The neoplastic proliferation was negative for cytokeratin (CK, AE1/AE3), epithelial membrane antigen (EMA), leukocyte common antigen (LCA), human melanoma black-45 (HMB45), Melan A, CD30, CD117, and discovered on GIST-1 (DOG-1). Synaptophysin (Syn) and microtubule-associated protein-2 (MAP-2) were diffusely positive for epithelioid cells and highlighted the ganglion-like cells (**Figure 3A**). The staining pattern of chromogranin A (CgA) was similar, whereas the number and intensity were limited (**Figure 3B**). The ganglion-like cells stood out in neurofilament (NF) staining (**Figure 3C**). Neu-N was negative in both epithelioid and ganglion-like cells. Progesterone receptor (PR) was positive in some epithelioid cells, whereas estrogen receptor (ER) was negative (**Figure 3E**). S-100 and SOX-10 were positive in the spindle cell proliferation and around the epithelioid cells (**Figure 3D**). CD34 was positive in the spindle cells and endothelia. Smooth muscle actin (SMA) and desmin stained the muscularis mucosa, which confirmed that the tumor infiltrated the mucosa (**Figure 3F**).

Integrating morphology and immunostaining, GP was the permanent pathological diagnosis. The patient received no adjuvant therapy and remained with no recurrence in 2 years’ follow-up.

## Discussion

GP is a rare neuroendocrine tumor (NET), and up to 300 cases have been reported until now. GPs affect individuals ranging from 15 to 84 years old with a mean of about 53 years old and are a little more prevalent in males with a male-to-female ratio of 1.5:1 (1). Nearly 90% of GPs were documented to be located in the duodenum, and involvement of other sites such as spinal cord, respiratory system, and digestive tract was also reported (2).

The presenting symptoms and complaints of GPs in digestive tracts include gastrointestinal bleeding, abdominal pain, anemia, and so on, which have no reliable diagnostic signs. Imaging examinations often demonstrate a mass lesion (3–5).

GPs range in size from 0.5 to 10 cm with an average of 2.5 cm in maximum diameter. In pathological investigation, they are always well-circumscribed and non-encapsulated, whereas some cases are infiltrative focally or even extensively. GPs in the duodenum are located in the submucosa, expanding to adjacent lamina propria or muscularis propria (2). Therefore, preoperative pathological diagnosis is difficult through endoscopic biopsy due to a relatively deep location, and definite diagnosis requires resection of the mass (6).

GP consists of three distinct cellular elements, including the epithelioid, ganglion-like, and spindle cells (7). The epithelioid cells have eosinophilic abundant cytoplasm with a round nucleus. They arrange in nest or zellballen pattern. They are positive for Syn and CgA, around which S-100 and SOX-10 are positive. They resemble PGL in cytological, architectural, and immunostaining features. Compared with the epithelioid cells, the ganglion-like cells are even larger and have more prominent nucleoli. They are always scattered or sometimes merged with the epithelioid or spindle cells individually or in small clusters. The immunophenotypes of the ganglion-like cells are similar with those of the epithelioid cells, whereas NF is positive uniquely. The bland spindle cells arrange in fascicular clusters and are positive for S-100 and SOX-10, which resemble neurofibroma. The neoplasm did not display mitosis and necrosis.

The proportion of the three cellular types is variable. In tumors predominant of spindle cells, the differential diagnosis includes spindle cell neoplasms, such as schwannoma and gastrointestinal stromal tumor (GIST) (8). The presence of epithelioid and ganglion-like cells, although perhaps rare, is the most important clue against schwannoma. Negative immunohistochemical expression for DOG-1 and CD117 provides compelling evidence against GIST.

In tumors predominant of ganglion-like or epithelioid cells, the differential diagnosis includes epithelial tumors, melanoma, and well-differentiated neuroendocrine neoplasms (NENs). The first two tumors are excluded by immunostaining of epithelial or melanic markers with relative ease.

The relationship between GPs and NENs is still in argument (9, 10). World Health Organization (WHO) classification of digestive system updated the classification and grading criteria for NENs in 2019. In the fourth edition, proliferation activity was nearly the only criterion, and NENs were classified into well-differentiated NETs including G1 and G2 and poorly differentiated neuroendocrine carcinomas (NECs) as G3. In the fifth edition, morphological characteristics such as atypia or necrosis are more emphasized besides proliferation activity, thus NETs and NECs are first distinguished. NETs are graded as G1, G2, and G3 according to proliferative activity. NECs are subtyped as small-cell NECs and large-cell NECs. In brief, the most significant difference between the two classifications is that NET G3 was considered to be synonymous with poor differentiation (i.e., NECs) in the fourth edition, while NET G3 and NECs are distinguished now. In addition, the cutoff and counting methods of proliferation index are adjusted. GPs have been classified among well-differentiated NETs by some authors (11). However, diversity of GPs is different from relative consistence of NETs in morphology. More significantly, GPs have a more indolent clinical behavior and favorable prognosis than NETs (12). Thus, in our opinion, GP is supposed to be differentiated from NET G1.

A multidisciplinary team (MDT) involving endoscopy, digestion, imaging, and pathology is always assembled for NENs in the gastrointestinal tract. An important clinical distinction among NENs relates to their hormonal functionality. Functions are determined by the abnormal production of hormones by NENs. Clinical non-functioning NENs may also produce hormones that do not result in clinical symptoms. Rare cases of GP were reported to have hormonal functions (13). In a sense, NENs in the duodenum can be subtyped into non-functioning NET, gastrinoma, somatostatinoma, enterochromaffin-cell carcinoid, NEC, and GP.

Entity of GPs remains a problem. As early as 2005, some authors pointed out that histological differences between pheochromocytoma-ganglioneuromas and GPs were not clear (14). Actually, GPs can be subdivided into two neoplastic components including PGL and ganglioneuroma. The occurrence of two or more synchronous tumors, which admixed so intimately with each other as to be impossibly separated topographically, is supposed to be named for composite tumors. Therefore, GP is preferred as paraganglioma-ganglioneuroma, a subtype of composite PGLs. It is noteworthy that immature ganglion cells are supposed to be explored to exclude the possibility of paraganglioma-neuroblastoma, especially in young patients.

*Succinate dehydrogenase (SDH)* is known as a tumor suppressor gene that plays a role in PGL (15). *SDH* is involved in the mitochondrial tricarboxylic acid (TCA) cycle and composed of four subunits: *SDHA*, *SDHB*, *SDHC*, and *SDHD*. Inactivating mutations in the *SDH* genes contribute to PGL, and mutations in *SDHB* are the most frequent among the four subunits (16). It is not known whether mutations of *SDH* are involved in GPs due to rare cases. It is a pity that *SDH* was not studied in the case.

The novel expression of PR, which was also confirmed by some other recent studies, is worthy of attention (2). PR is supposed to be an alternative marker for differential diagnosis. GP is postulated to be originated from pancreas islet remnant or ectopia, since pancreatic islet cells also express PR (2). GP is always located in the submucosa, inducing hypothesis of origin from Meissner plexus (17). Origin of GP needs to be elucidated.

Although the clinical behavior of GP is usually benign, up to 10% of cases occurred with regional lymph node metastasis and only a few cases occurred with distant metastasis to bone, liver, or pelvic cavity (18–21). Age, tumor size, and depth of invasion appear to be related to metastasis (2). The immunohistochemical prognostic factors in NETs, such as Ki-67, P53, and Bcl-2, are not indicative of malignant potential. It is noted that all the three cellular components are supposed to be present in metastasis. The patients with metastasis have favorable prognosis with long survival periods, whereas only one case followed an aggressive clinical course and died of the disease (21). Endoscopic resection of duodenal GP appears enough for most cases, while additional surgery needs to be managed in cases of positive margins (22, 23). Adjuvant chemotherapy and irradiation appear to have no effect, even in cases with metastasis.

In conclusion, we presented a rare case of 51-year-old man with GP as a polyp in the descending portion of the duodenum. Pathological examination showed that a neoplasm was predominantly located in the submucosa and infiltrated the lamina propria. The tumor was composed of epithelioid, ganglion-like, and spindle cells. Syn, MAP-2, and CgA were positive in the epithelioid and ganglion-like cells in variety, and NF staining highlighted the ganglion-like cells. S-100 and SOX-10 were positive in the spindle cell proliferation and around the epithelioid cells. PR was also positive in the epithelioid cells. Origin of GP is presumed to be related to pancreas islet. GP is supposed to be distinguished from NET G1 and designated as paraganglioma-ganglioneuroma, a kind of composite PGL. Most GPs displayed benign clinical biological behaviors, and a few occurred in regional lymph nodes or distant metastasis. A large majority of cases follow a favorable prognosis, even with metastasis.

## Data Availability Statement

The raw data supporting the conclusions of this article will be made available by the authors without undue reservation.

## Ethics Statement

The studies involving human participants were reviewed and approved by the ethics committee of the 7th Medical Center, Chinese PLA General Hospital. The patients/participants provided their written informed consent to participate in the study.

## Author Contributions

JL collected information and wrote the article. L-PW made the pathological diagnosis. P-SZ performed histological and immunohistochemical investigation. All authors contributed to the article and approved the submitted version.

## Conflict of Interest

The authors declare that the research was conducted in the absence of any commercial or financial relationships that could be construed as a potential conflict of interest.

## Publisher’s Note

All claims expressed in this article are solely those of the authors and do not necessarily represent those of their affiliated organizations, or those of the publisher, the editors and the reviewers. Any product that may be evaluated in this article, or claim that may be made by its manufacturer, is not guaranteed or endorsed by the publisher.
